# Investigation of the cytokine response to NF-κB decoy oligonucleotide coated polysaccharide based nanoparticles in rheumatoid arthritis *in vitro* models

**DOI:** 10.1186/s13075-015-0824-x

**Published:** 2015-11-04

**Authors:** Patricia R. Wardwell, Martin B. Forstner, Rebecca A. Bader

**Affiliations:** Syracuse Biomaterials Institute, Syracuse University, 318 Bowne Hall, Syracuse, NY 13244 USA; Department of Biomedical and Chemical Engineering, Syracuse University, 121 Link Hall, Syracuse, NY 13244 USA; Department of Physics, Syracuse University, Syracuse, NY 13244 USA

**Keywords:** Rheumatoid arthritis, Nanoparticles, NF-κB, Immunomodulation, Primary cells

## Abstract

**Introduction:**

The transcription factor nuclear factor-kappa B (NF-κB) is highly involved in regulation of a number of cellular processes, including production of inflammatory mediators. Thus, this transcription factor plays a role in pathology of many diseases, including rheumatoid arthritis, an autoimmune disease hallmarked by an imbalance of pro and anti-inflammatory cytokines. Small nucleic acids with sequences that mimic the native binding site of NF-κB have been proposed as treatment options for RA; however due to low cellular penetration and a high degree of instability, clinical applications of these therapeutics have been limited.

**Methods:**

Here, we describe the use of N-trimethyl chitosan-polysialic acid (PSA-TMC) nanoparticles coated with decoy oligodeoxynucleotides (ODNs) specific to transcription factor NF-κB (PSA-TMC-ODN) as a method to enhance the stability of the nucleic acids and facilitate increased cellular penetration. In addition to decoy ODN, PSA-TMC nanoparticles were loaded with RA therapeutic methotrexate (MTX), to assess the anti-inflammatory efficacy of a combination therapy approach. Two different in vitro models, a cell line based model as well as a primary RA cell model were used to investigate anti-inflammatory activity. One way ANOVA followed by Holm-Sidak stepdown comparisons was used to determine statistical significance.

**Results:**

In general, free ODN did not significantly affect secretion of pro-inflammatory cytokines interleukin-6 (IL-6) and interleukin-8, (IL-8) while free MTX had variable efficacy. However, PSA-TMC-ODN and PSA-TMC-ODN-MTX resulted in significant decreases in the inflammatory mediators IL-6 and IL-8 in both cell models. In addition, PSA-TMC exhibited sufficient cellular uptake, as observed through fluorescence microscopy.

**Conclusions:**

These results support our previous findings that PSA-TMC nanoparticles are an effective delivery vehicle for small nucleic acids, and effectively alter the pro-inflammatory state characteristic of RA.

## Introduction

Rheumatoid arthritis (RA) is an autoimmune disease characterized by inflammation of the synovial tissue of joints. Over time, the infiltration of immune cells to the synovial lining leads to hyperplasia, increased vascular growth, and formation of a tumor-like tissue known as the pannus [1]. The physiology of a chronic inflammatory state eventually results in cartilage degradation and bone resorption. An imbalance of proinflammatory and anti-inflammatory cytokines contributes to the state of chronic inflammation. Briefly, the levels of anti-inflammatory cytokines (interleukin (IL)-4, IL-10, and IL-13) present in the synovium are too low to combat the effects of proinflammatory cytokines (tissue necrosis factor alpha (TNFα), IL-1, IL-6, and IL-8) [2]. Of the cells present in the RA synovial lining, “macrophage-like” cells and activated synovial fibroblasts are accepted as the primary mediators of the proinflammatory/anti-inflammatory imbalance [3, 4].

**Fig. 1:**
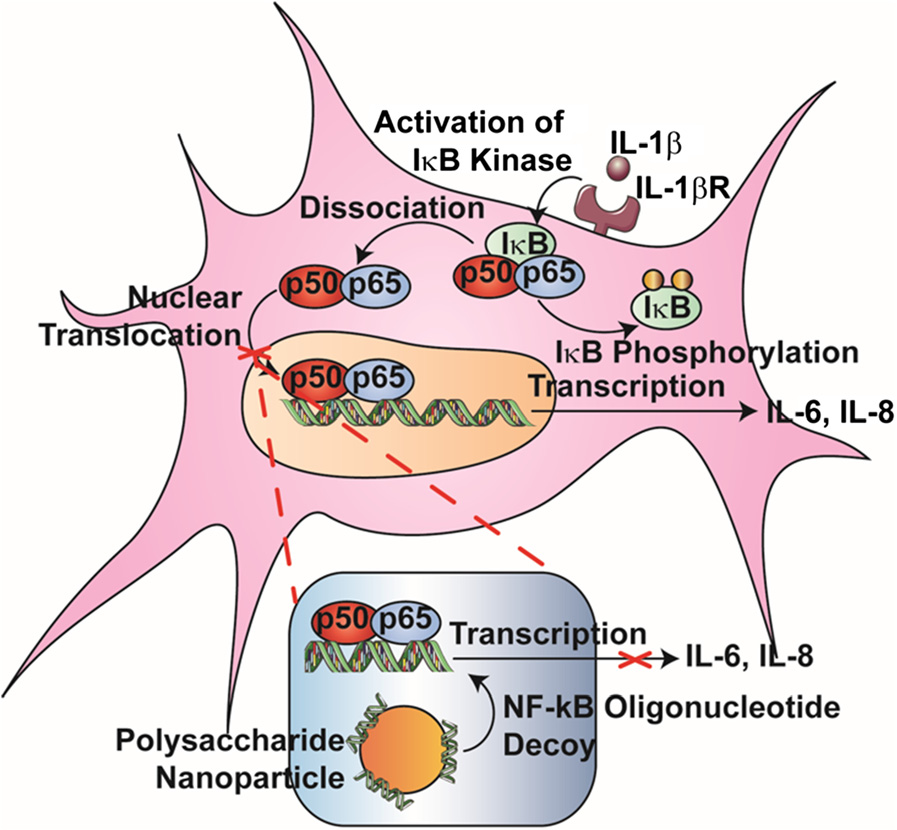
NF-κB pathway activation in a RA synovial fibroblast cell. Proinflammatory cytokines, including IL-1β, activate the cell signaling pathway associated with NF-κB, including activation of IκK, phosphorylation and inactivation of Iκβ, and translocation of NF-κB to the nucleus. NF-κB decoy ODNs can prevent translocation of the transcription factor, as well as subsequent transcription of NF-κB-dependent genes. *IκB* inhibitor of NF-κB, *IL* interleukin, *NF-κB* nuclear factor kappa-light chain enhancer of activated B cells

Nuclear factor kappa-light chain enhancer of activated B cells (NF-κB) is a transcription factor involved in the regulation of a variety of cellular processes, including growth, apoptosis, and inflammatory and immune responses [5]. NF-κB-dependent gene expression is known to play a critical role in the observed cytokine imbalance, as well as to contribute to increased inflammation in RA [6, 7]. Under normal conditions, NF-κB is sequestered in the cytoplasm by means of a bound inhibitor known as inhibitor of NF-κB (IκB). External stimulation from inflammatory mediators, including IL-1β, leads to a signaling cascade that results in phosphorylation of the inhibitor, followed by dissociation of the NF-κB/IκB complex and subsequent nuclear translocation of NF-κB. Once inside the nucleus, NF-κB initiates transcription of proinflammatory cytokines, including IL-6 and IL-8, two cytokines highly involved in regulating inflammation in RA. IL-6 and IL-8 both possess NF-κB binding sites on their promotor regions, indicating they are highly regulated by NF-κB [8]. Transcription factor decoy oligonucleotides (ODNs) have the potential to reduce inflammation in RA by binding to NF-κB in the cytoplasm, preventing nuclear translocation, and mitigating transcription of proinflammatory proteins. The mechanism of decoy ODN is illustrated in Fig. 1.

Transcription factor decoy ODNs mimic the native DNA binding site of the transcription factor, but are only ~20 base pairs long and do not encode any genes. NF-κB decoy ODNs are desirable drug candidate because they provide a way to selectively regulate specific genes [9]. A number of reviews herald decoy ODNs as a potential future treatment for a variety of pathologies, including inflammatory and autoimmune disorders, such as RA [9–12]. However, despite promising potential for treatment, applications have been limited by low cellular penetration and a lack of stability of the nucleotides, which combined result in low overall bioavailability [13, 14]. To overcome stability problems, chemical modifications such as phosphorothioate or methyl phosphonate are often applied to the nucleotide backbones [15]. While these modifications enhance stability, they do not necessarily lead to increased delivery efficiency, resulting in the need for a high dose and frequently repeated delivery. This is not a sustainable method for delivery, as phosphorothioate nucleic acids have been shown to have a concentration-dependent toxicity [16, 17].

Methods such as viral vectors, cationic lipid formulations, and, more recently, cationic polymer formulations exist to overcome the barriers to nucleic acid delivery. In general, drug delivery systems for nucleic acids must have the following attributes: biocompatibility and biodegradability, reticuloendothelial system (RES) avoidance, nonimmunogenicity, cellular uptake capability, and cell or tissue specificity [18]. Viral vectors are associated with major drawbacks, including viral-induced immunogenicity, toxicity, mutation of the nucleic acid of interest with the viral DNA, and the potential for inactivation of the gene of interest due to recombination [19]. Lipofectamine reagents, marketed by Life Technologies (Carlsbad, CA, USA), have become the most referenced cationic lipid-based transfection reagent, with the claim of increased efficiency over other available reagents. However, these compounds often exhibit cytotoxicity and are prone to accumulation within the liver in vivo, leading to significant nucleic acid payload degradation [20, 21]. Recent research has focused on the alternative use of cationic polymers for nucleic acid delivery. Poly(ethyleneimine) (PEI) is one of the most widely used cationic polymers for nucleic acid delivery; however, PEI exhibits considerable toxicity toward a variety of mammalian cells [22, 23]. Delivery systems for RA, where the ultimate goal is to reduce inflammation, require materials that do not contribute to inflammation or the immune response and that exhibit low levels of toxicity. Therefore, an alternative delivery method to viral vectors, cationic lipids, and synthetic cationic polymers is needed.

We have recently described a nanoparticle system based on two natural polymers, *N*-trimethyl chitosan (TMC) and polysialic acid (PSA). This nanoparticle system is noncytotoxic, is nonimmunogenic, and has been shown to effectively deliver encapsulated disease-modifying anti-rheumatic drugs (DMARDs) and surface-coated NF-κB decoy ODNs when applied to in vitro models of RA and cystic fibrosis (CF), respectively [24, 25]. In this manuscript, we report the use of PSA–TMC nanoparticles (NP) as a delivery system to combine treatment of a DMARD, methotrexate (MTX), and NF-κB decoy ODN.

## Methods

### Materials

PSA (colominic acid) was obtained from Nacalai USA, Inc. (San Diego, CA, USA). TMC was produced via quaternization of chitosan (molecular weight 100–300 Da) obtained from Acros Organics (Morris, NJ, USA), as described previously by Sieval et al. [26]. Sodium tripolyphosphate (TPP) was purchased from Acros Organics. An NF-κB decoy ODN kit containing NF-κB decoy ODN (5′-CCT TGA AGG GAT TCC CTT CC-3′) and a scrambled ODN (SCO; 5′-TTG CCG TAC CTG ACT TAG CC-3′) was purchased from CosmoBio (Tokyo, Japan). Recombinant human IL-1β was obtained from R & D systems (Minneapolis, MN, USA). MTX was purchased from Enzo Life Sciences (Rochester, NY, USA). Alexa Fluor 488 succinimidyl ester was purchased from Invitrogen/Life Technologies (Grand Island, NY, USA).

### Cell culture

SW982 cells were obtained from ATCC (Manassas, VA, USA) and grown in Dulbecco’s modified Eagle’s medium (DMEM; Fisher Scientific, Pittsburgh, PA, USA) supplemented with 10 % fetal bovine serum (FBS; Atlanta Biologicals, Atlanta, GA, USA) until confluent. Primary RA cells were isolated from synovial tissue obtained from two Caucasian RA patients, both women between the ages of 50 and 59. The use of human samples was approved by the Syracuse University Institutional Review Board (IRB). The tissue samples were obtained by Dr Timothy Damron at Community General Hospital (Syracuse, NY, USA) following written and informed consent by each patient, as required by an IRB-approved protocol. Tissue was isolated following a protocol outlined by Zimmerman et al. [27]. Briefly, the synovial tissue was minced finely and incubated at 37 °C with 0.1 % Trypsin (Invitrogen, Carlsbad, CA, USA) in phosphate-buffered saline (PBS) for 30 minutes. Tissue was then digested for 2 hours in DMEM with 0.1 % Collagenase P. After digestion, tissue was filtered through a 100 μM filter. The resultant solution was centrifuged, and the pelleted cells were resuspended in DMEM with 10 % FBS, placed in a T-75 flask, and cultured at 37 °C with 5 % CO_2_. After three passages, the cells were stained with CD44-FITCmAB (Santa Cruz Biotechnology, Santa Cruz, CA, USA) to confirm fibroblast cells. To supplement the two sets of primary cells obtained through synovial tissue isolation, human fibroblast-like synoviocytes (HFLS; lot numbers 2884 and 2956, female Caucasian) were obtained from Cell Applications (San Diego, CA, USA) and cultured in DMEM with 10 % FBS at 37 °C with 5 % CO_2_.

### Nanoparticle preparation and characterization

ODN-coated NP were prepared as described previously [25]. Briefly, 6.4 mg TMC (55 % quaternization) were dissolved in 3.0 ml of 0.3 % acetic acid in a glass vial. Meanwhile, 3.2 mg PSA and 1.0 mg TPP were dissolved in 2.0 ml DI (Deionized) water. To prepare nanoparticles loaded with MTX, 2.4 mg MTX were added to the aqueous PSA solution. The latter solution was sonicated for 10 minutes and then added drop-wise to the TMC solution with stirring. Stirring was continued at room temperature for 20 minutes. At this time, 10 μg ODN were added to the nanoparticle suspension. Stirring was continued for an additional 10 minutes to ensure uniform electrostatic adhesion of ODN to the nanoparticle surface, as well as complete dispersion. Upon completion of stirring, centrifugation at 3000 rpm for 15 minutes yielded a pellet of ODN-coated NP.

Nanoparticle size, zeta potential, and polydispersity index were determined using a Malvern Zetasizer NanoZS90 (Malvern Instruments, Malvern, UK). Following centrifugation, nanoparticles were resuspended at a concentration of 2 mg/ml in DI water and filtered through a 0.45 μM syringe filter. Samples were loaded into cuvettes or capillary cells for measurements at 25 °C.

### Determination of MTX loading

High-performance liquid chromatography (HPLC) was used to determine the amount of MTX loaded into ODN-coated nanoparticles. After nanoparticles were pelleted via centrifugation, supernatant samples were saved and analyzed using a Prominence Ultrafast Liquid Chromatography System (Shimadzu Instruments, Kyoto, Japan). Samples were run using a 93:7 (v/v) mixture of 50 mM ammonium acetate and acetonitrile mobile phase at a flow rate of 0.75 ml/minute with a 100 μl injection volume. The detection wavelength used was 210 nm. To determine the amount of MTX present based on peak area, a calibration curve of eight known concentrations (from 50 to 0.39 μg/ml) of MTX was constructed. PeakFit 4.2 (SYSTAT Software, San Jose, CA, USA) software was used to analyze the peak area.

### In vitro efficacy of ODN-coated NP

SW982 cells or primary RASF cells were plated on 24-well plates at a density of 20,000 cells/well. Nanoparticles were prepared as described in Nanoparticle preparation and characterization. In addition to ODN-coated nanoparticles (NP-ODN), bare nanoparticles (NP), MTX-loaded nanoparticles (NP-MTX), ODN-coated MTX-loaded nanoparticles (NP-MTX-ODN), and nanoparticles coated with a SCO (NP-SCO) were prepared. As a control, MTX alone was prepared at a concentration of 1.0 mg/ml DMEM media. After centrifugation, all nanoparticles were resuspended in serum-free DMEM at a concentration of 1.0 mg/ml. Then 500 μl of each treatment group was added to the 24-well plate in duplicate as follows: media alone (control); ODN alone; NP-ODN; NP; NP-SCO; NP-MTX; NP-ODN-MTX; and MTX alone. The complexes were removed and media was replaced after 4 hours to allow for normal growth conditions. The amount of ODN in each treatment group was held constant at 1 μg/ml, or approximately 500 ng/well. This concentration does not have an impact on cellular proliferation. Twenty-four hours after initial complex addition, inflammation was induced with the addition of 1.0 ng/ml IL-1β. This concentration has been shown to increase levels of IL-6 and IL-8 when administered to SW982 cells [28]. After incubation at 37 °C for an additional 24 or 48 hours, supernatant samples were collected and stored at –80 °C for analysis of IL-6 and IL-8.

### Quantitative analysis of inflammatory cytokines

Enzyme-linked immunosorbent assay (ELISA) kits for IL-6 and IL-8 were purchased from Peprotech (Rocky Hill, NJ, USA) and run according to the manufacturer’s instructions. Samples were run in duplicate, and each experiment was repeated independently at least three times.

### In vitro cellular uptake

To examine internalization of the nanoparticles, cellular uptake experiments were performed. Prior to nanoparticle synthesis, TMC was tagged with Alexa-Fluor 488 carboxylic acid, succinimidyl ester, and mixed isomers in dimethylsulfoxide (DMSO; 1 mg/ml) (Invitrogen, Grand Island, NY, USA). Then 25 mg TMC were dissolved in 4 ml of 0.1 M sodium bicarbonate buffer (pH 8.3), 500 μl AF 488 dye were added, and the solution was stirred for 1 hour at room temperature. Upon completion of stirring, the resultant material was dialyzed for 48 hours against water to ensure removal of unreacted dye. In addition, the amount of TMC used has an excess of reactive amine groups relative to amount of Alexa Fluor 488, and therefore the amount of unreacted dye was expected to be negligible. ^1^H-NMR confirmed dye conjugation. Cells were plated on lysine-coated glass-bottom dishes (Mattek Corp, Ashland, MA, USA) at a density of 100,000 cells per dish 2 days prior to scheduled imaging to allow for adherence and confluence. ODN-coated NP were prepared using Alexa Fluor 488 tagged TMC, and Texas Red tagged ODN. On the day of imaging, sterile filtered NP, NP-ODN, and ODN alone were administered to the plated cells at a concentration of 1.0 mg/ml, 1.0 mg/ml, and 500 ng/ml, respectively. The concentrations used were well below the concentrations associated with cytotoxicity in order to avoid any changes in uptake due to activation of a cellular inflammatory response. Complexes were incubated with the cells for 45 minutes at 37 °C prior to removal. The cells were washed three times with 1× PBS and imaged using a Nikon Eclipse Ti (Nikon Metrology, Brighton, MI, USA) inverted microscope.

### Statistical analysis

IL-6 and IL-8 protein levels were expressed relative to an untreated, stimulated control group, with all data presented as mean ± standard deviation (SD) for all groups (*n* ≥3). One-way analysis of variance (ANOVA) followed by Holm–Sidak testing for multiple comparisons was performed to compare IL-6 and IL-8 protein secretion following treatment and inflammatory stimulation. All statistical tests were conducted with α = 0.05.

## Results

### Nanoparticle loading and characterization

Our laboratory previously reported the use of NP for effective delivery of conventional, small molecule therapeutics, such as MTX and dexamethasone, as well nucleic acid-based therapeutics, particularly decoy ODN [24, 29]. As expected based on these prior studies, NP possessed a size of close to 100 nm (115 nm) and a positive zeta potential (37 mV), while NP-ODN nanoparticles possessed a significantly larger size with a diameter of 159 ± 15 nm and a decrease in surface charge to 23 mV [24, 25]. Furthermore, NP-ODN loaded with MTX led to another slight size increase, insignificantly larger than NP-ODN alone, with a diameter of 184 ± 5.6 nm while maintaining a positive zeta potential of approximately 33 ± 6.5 mV. All nanoparticle formulations possessed size between 100 and 200 nm, favorable for evading the RES in applications for drug delivery [30].

HPLC was performed to determine the amount of MTX loaded within the NP-ODN-MTX. Loading capacity and loading efficiency values of 0.20 mg MTX/mg nanoparticle and 86.7 %, respectively, were obtained. The high loading efficiency of MTX suggests that the majority of the drug is encapsulated prior to addition of ODN to the nanoparticle suspension, and therefore interactions between ODN and MTX are limited.

### Effect of ODN-loaded and MTX-loaded NP on IL-6 and IL-8 secretion in RA in vitro models

Previously, we have reported the use of a luciferase reporter assay to confirm that the reduction in inflammatory protein levels was in fact occurring due to decoy ODN interference with NF-κB [24]. In the current study, to initially determine efficacy of NF-κB decoy ODN-loaded and MTX-loaded NP, the SW982 cell line was used as a model of RA. The SW982 cell line has been shown to mimic activated RA synovial fibroblast cells with regards to the expression of inflammatory mediators, particularly when stimulated in 1 ng/ml IL-1β [31]. We have previously conducted cytotoxicity studies of NP formulations and concluded that NP, as well as NP-ODN, NP-MTX, and MTX alone, do not impact cellular proliferation at low concentrations and are therefore appropriate for this study [24, 25]. In addition to results on cytotoxicity, we recently demonstrated that NP-ODN are effective at reducing inflammation in an in vitro model of CF [24].

To assess the bioactivity of NP coated with the NF-κB decoy ODN and/or loaded with MTX, the secretion of two potent inflammatory mediators, IL-6 and IL-8, by SW982 cells was investigated. Both of these proinflammatory mediators are directly influenced by NF-κB and play a major role in the inflammatory response in RA. IL-6 is a multifunctional cytokine, with the ability to regulate the immune response, inflammation, and hematopoiesis, and plays a crucial role in RA pathogenesis [32]. IL-8 was chosen as a representative chemokine and is responsible for recruiting immune cells to the synovium and contributing to the tumor-like pannus tissue. In addition, IL-8 is involved in upregulation of inflammation via paracrine signaling mechanisms in the RA synovium [33]. The mechanism of action of MTX in RA treatment and inflammatory activity is currently unresolved; however, the drug is believed to interfere with cell folate metabolism. Furthermore, several reports suggest MTX acts on NF-κB as an inhibitor [34]. We explored coadministration of NF-κB decoy ODN and MTX to observe any potential synergistic activity. IL-6 and IL-8 levels were examined in response to treatment with NP-ODN, NP-MTX, NP-ODN-MTX, ODN alone, and MTX alone using immunoassays.

**Fig. 2:**
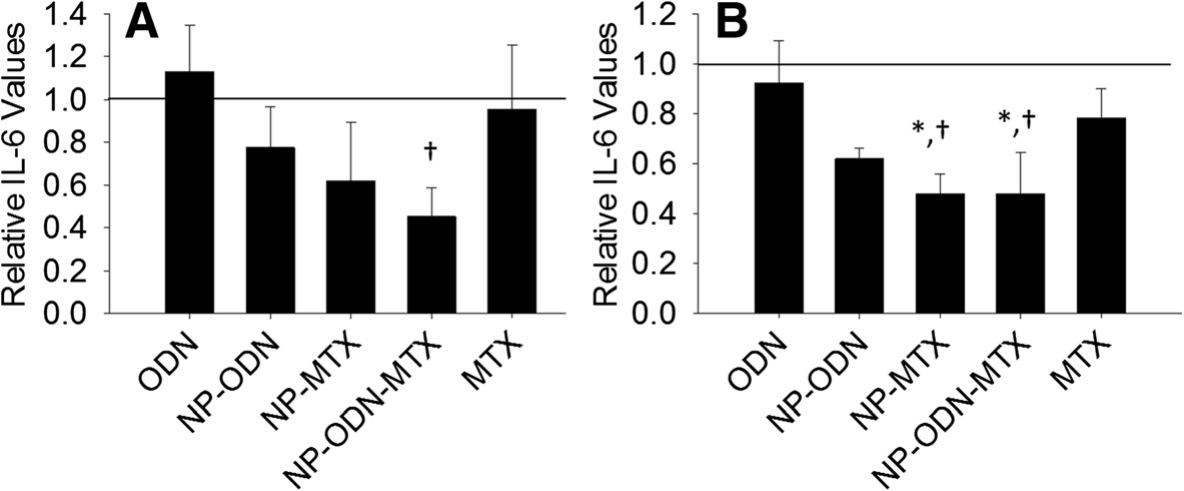
ELISA was performed to determine levels of IL-6 secretion by SW982 cell line RA model cells, after treatment with ODN alone, NP-ODN, NP-MTX, NP-ODN-MTX, and MTX alone, and stimulation with IL-1β at (**a**) 24 hours and (**b**) 48 hours. Results are expressed as fold changes of IL-6 levels relative to an untreated control (*solid line* at 1). One-way ANOVA followed by Holm–Sidak multiple comparisons testing was used to determine the impact of treatment on IL-6 secretion. *Significant difference from the control. †Significant difference from ODN alone. Data presented as mean ± SD (*n* = 3). *IL* interleukin, *MTX* methotrexate, *NP* PSA-TMC nanoparticles, *ODN* oligonucleotide

The IL-6 secretion profile in response to different treatment groups is shown in Fig. 2. In general, we were interested in the IL-6 response of cells subjected to the different treatment groups in comparison with untreated control cells and in comparison with cells treated with ODN alone. At 24 hours (Fig. 2a), cells treated with NP-ODN-MTX displayed a significant reduction of IL-6 relative to untreated control cells. A decrease in IL-6 levels in comparison with untreated control cells was also observed following treatment with NP-ODN; however, this reduction was not great enough to be significant. At 48 hours (Fig. 2b), a significant decrease relative to both untreated control cells and cells administered ODN alone was observed following treatment with NP-MTX and NP-ODN-MTX. NP-ODN displayed trends at 48 hours similar to those at 24 hours. This treatment resulted in a decrease in IL-6 levels in comparison with both untreated control cells and ODN alone; however, the decrease was not great enough to be significantly different.

**Fig. 3:**
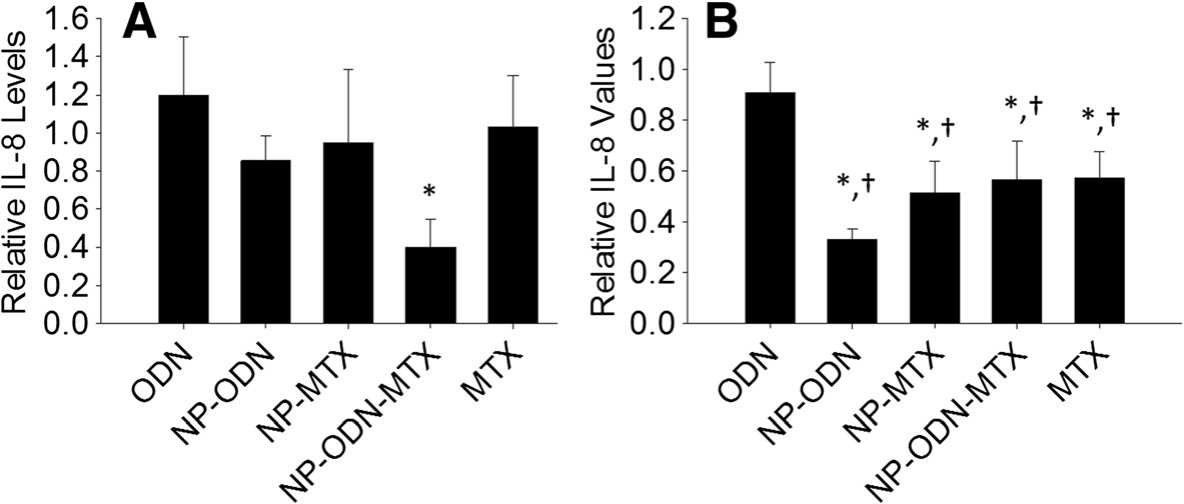
ELISA was performed to determine levels of IL-8 secretion by SW982 cell line RA model cells, after treatment with ODN alone, NP-ODN, NP-MTX, NP-ODN-MTX, and MTX alone, and stimulation with IL-1β at (**a**) 24 hours and (**b**) 48 hours. Results are expressed as fold changes of IL-8 levels relative to an untreated control (*solid line* at 1). One-way ANOVA followed by Holm–Sidak multiple comparisons testing was used to determine the impact of treatment on IL-8 secretion. *Significant difference from the control. †Significant difference from ODN alone. Data presented as mean ± SD (*n* = 3). *IL* interleukin, *MTX* methotrexate, *NP* PSA-TMC nanoparticles, *ODN* oligonucleotide

The IL-8 secretion profile from SW982 cells in response to treatment with different nanoparticle formulations is portrayed in Fig. 3. After 24 hours (Fig. 3a), NP-ODN-MTX resulted in a significant decrease in IL-8 levels compared with ODN administered alone. While the level of IL-8 in response to NP-ODN-MTX treatment was lower than the untreated control, the difference was not significant. At 48 hours (Fig. 3b), multiple significant decreases in IL-8 levels were observed. Cells treated with NP-ODN, NP-MTX, NP-ODN-MTX, and MTX alone all had IL-8 levels significantly lower than the untreated control cells and cells treated with ODN alone.

These results suggest that NP can be used to deliver decoy ODN and MTX, alone and simultaneously, to activated RA synovial fibroblasts. To further validate the ability of NP to serve as an effective treatment strategy for RA, in vitro experiments were also conducted with primary cells. Previous reports have noted discrepancies in cytokine production between cell line models and primary RASF cells [35]. Furthermore, a literature search revealed that immortalized cells, such as SW982, constitutively express the NF-κB pathway, indicating that they may be more susceptible to NF-κB interference than primary RASF cells [36].

**Fig. 4:**
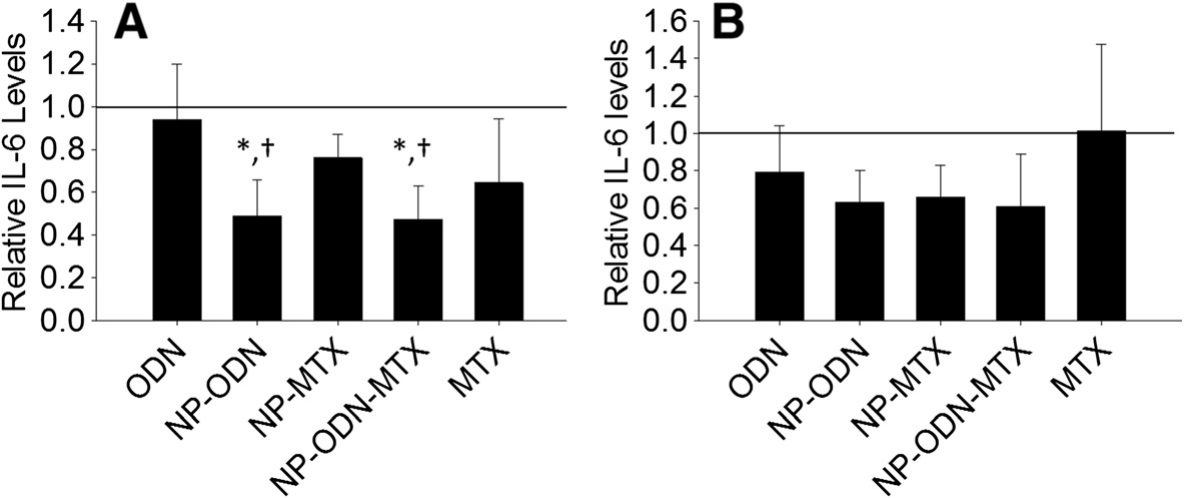
ELISA was performed to determine levels of IL-6 secretion by primary RA synovial fibroblast cells, after treatment with ODN alone, NP-ODN, NP-MTX, NP-ODN-MTX, and MTX alone, and stimulation with IL-1β at (**a**) 24 hours and (**b**) 48 hours. Results are expressed as fold changes of IL-6 levels relative to an untreated control (*solid line* at 1). One-way ANOVA followed by Holm–Sidak multiple comparisons testing was used to determine the impact of treatment on IL-6 secretion. *Significant difference from the control. †Significant difference from ODN alone. Data presented as mean ± SD (*n* = 4). *IL* interleukin, *MTX* methotrexate, *NP* PSA-TMC nanoparticles, *ODN* oligonucleotide

Primary RASF cell cytokine secretion was investigated following treatment with ODN, NP-ODN, NP-MTX, NP-ODN-MTX, and MTX. At 24 hours (Fig. 4a), a significant reduction in IL-6 secretion by the primary cells was observed in response to NP-ODN and NP-ODN-MTX in comparison with untreated control cells, as well as cells administered ODN alone. While cells treated with NP-MTX and MTX alone experienced a decrease in levels of IL-6, the decrease was not great enough to be considered significant. A lack of significant reduction of IL-6 secretion in response the NP-MTX and MTX alone is in accordance with several reports, described in further detail in the Discussion, stating that MTX does not have a direct effect on IL-6 levels in primary RASF cells. At 48 hours (Fig. 4b) although trends similar to those at 24 hours are seen with reductions in IL-6 levels in response to NP-ODN, NP-MTX, and NP-ODN-MTX, significant reductions were not observed in response to any NP treatment.

**Fig. 5:**
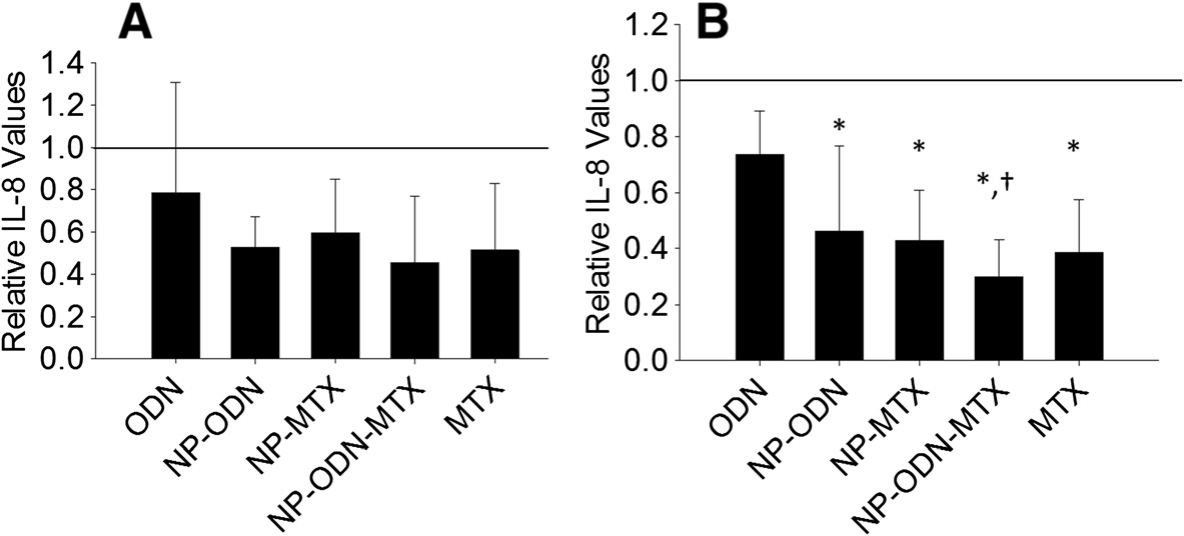
ELISA was performed to determine levels of IL-8 secretion by primary RA synovial fibroblast cells, after treatment with ODN alone, NP-ODN, NP-MTX, NP-ODN-MTX, and MTX alone, and stimulation with IL-1β at (**a**) 24 hours and (**b**) 48 hours. Results are expressed as fold changes of IL-8 levels relative to an untreated control (*solid line* at 1). One-way ANOVA followed by Holm–Sidak multiple comparisons testing was used to determine the impact of treatment on IL-8 secretion. *Significant difference from the control. †Significant difference from ODN alone. Data presented as mean ± SD (*n* = 4). *IL* interleukin, *MTX* methotrexate, *NP* PSA-TMC nanoparticles, *ODN* oligonucleotide

The IL-8 secretion response of primary RA cells to NP treatments is depicted in Fig. 5. At 24 hours (Fig. 5a), despite similar reduction trends to those observed for primary IL-6 secretion and SW982 IL-8 secretion, the differences were not significant. At 48 hours (Fig. 5b), NP-ODN, NP-MTX, NP-ODN-MTX, and MTX alone all resulted in significant decreases in IL-8 secretion when compared with the untreated control. Furthermore, NP-ODN-MTX treatment resulted in a significant decrease when compared with ODN alone.

### Cellular uptake of NP-ODN

**Fig. 6:**
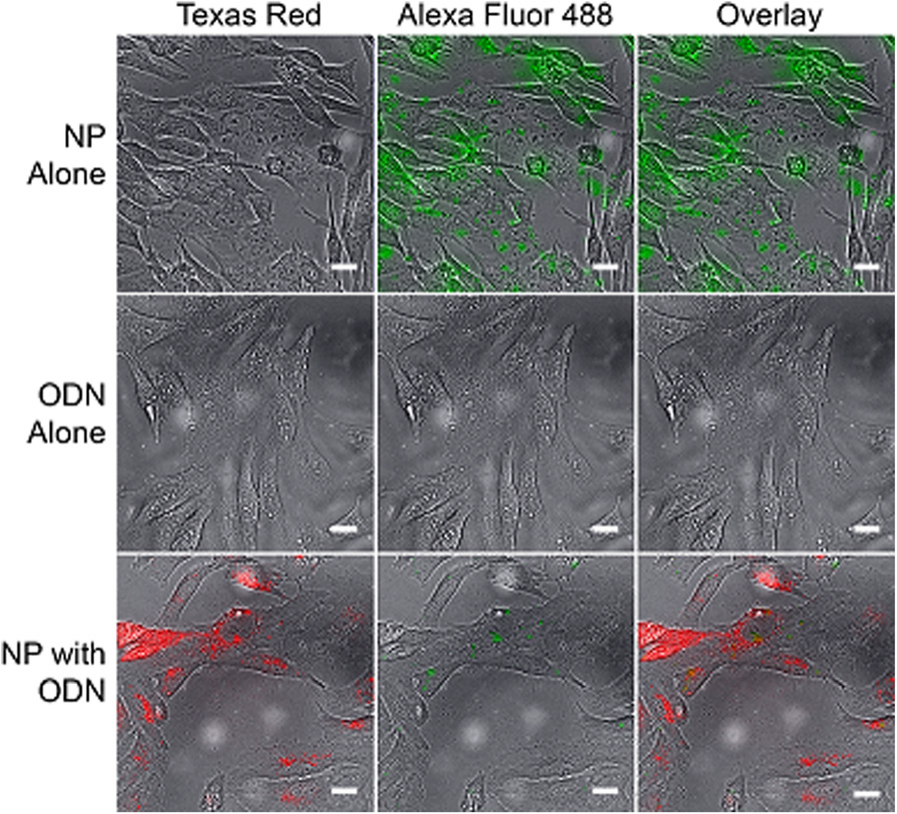
Cellular uptake visualization of Alexa Fluor 488 tagged PSA-TMC coated with Texas Red labeled decoy ODNs in SW982 synovial sarcoma cells. Cells were incubated with tagged particles for 45 minutes at 37 °C, and then imaged using an inverted fluorescent microscope. When administered alone, decoy ODN do not register a large signal. However, when delivered using the PSA-TMC carrier system, both red and green signals are clearly seen. *NP* PSA-TMC nanoparticles, *ODN* oligonucleotide

To facilitate visualization of carrier uptake and localization of NP and NPDON in vitro, TMC was modified with a green (Alexa Fluor 488) fluorescent tag, and ODN was modified with red (Texas Red). Tagged NP, NP-ODN, and ODN were incubated with SW982 cells at 37 °C for 45 minutes, and visualized. A composite image from the uptake experiments is shown in Fig. 6. NP-ODN demonstrated cellular uptake of both NP and ODN, while ODN alone did not appear to enter the cells.

## Discussion

A major barrier in the advancement of nucleic acid therapies to achieving clinical relevance is a general lack in ability of the negatively charged nucleic acid to enter the negatively charged cell membrane. A number of positively charged carrier systems and transfection reagents have been explored to overcome this barrier; however, many of these are associated with toxicity, immunogenicity, and/or are highly variable based on cell type [37]. The NP carrier system presented here has been found to be noncytotoxic, while maintaining a positive surface charge [24, 25, 38]. We expect that the positive surface charge of the particles due to TMC facilitates interaction with negatively charged cell membranes, increasing ODN cellular uptake. Meanwhile, PSA has no known receptors in the body, making it an optimal choice for a component, as this will probably allow for RES evasion and reduce the likelihood of inducing an immune response. Further, PSA has properties similar to polyethylene glycol (PEG), a polymer commonly used to extend circulation time via incorporation into nanocarrier systems or protein conjugates [25, 39–41]. In sum, we have reported herein a nanoparticle system based on natural polysaccharides, which is anticipated to reduce immunogenicity and enhance hydrophilicity. An NF-κB decoy ODN was chosen as the NA (Nucleid acid) drug of choice for this study due to the known activity of NF-κB in RA pathology.

Under normal conditions, NF-κB is bound to an inhibitor in the cytoplasm. However, in response to an inflammatory stimulus, the inhibitor undergoes phosphorylation, leading to dissociation of the NF-κB/inhibitor complex and subsequent nuclear translocation of the transcription factor. For this study, we chose to quantify IL-6 and IL-8 as the representative cytokine and chemokine, respectively. In addition to other roles in the inflammatory response, IL-6 and IL-8 play a role in stimulation of vascular endothelial growth factor (VEGF), a growth factor linked to production of blood vessels [42]. The newly and hence typically rapidly formed blood vessels have larger pore sizes between the endothelial barrier than normal blood vessels, which can be exploited for drug delivery via the enhanced permeation and retention (EPR) effect [43]. Colloidal carrier systems between 100 and 200 nm can passively accumulate in areas associated with blood vessels with larger, leaky pores in the endothelial barrier; thus, the EPR effect is a means of passive targeting [30]. While RA pathology does not exhibit the retention aspect of this phenomenon, the enhanced permeation appears to be great enough to act as a passive targeting mechanism [44, 45].

As NF-κB has a well-established involvement in RA, the transcription factor is an enticing target for drug candidates. However, as demonstrated here and in previous studies, administration of decoy ODNs alone results in low efficacy and delivery with many available reagents results in high degrees of cytotoxicity [24]. As shown in Fig. 6, the use of PSA–TMC as a carrier system for decoy ODNs drastically increases the presence of the decoy within the cell, proving an increased amount of therapeutic at the site of action while maintaining cell health. With the use of the PSA-TMC carrier system, a lower amount of ODN can be administered, thereby decreasing potential adverse effects from the decoys themselves.

We have previously established increased anti-inflammatory bioactivity of an NF-κB decoy when administered via NP in comparison with administration without a delivery vehicle in a CF in vitro model [24]. Likewise, in the current study, decoy ODN efficacy was increased when administered via PSA–TMC to primary and cell line in vitro models of RA. SW982 cells yielded significantly decreased levels of IL-6 in response to treatment with NP-ODN-MTX at 24 and 48 hours and in response to NP-MTX at 48 hours only. As primary cells are isolated from different individuals, it is not unexpected to observe increased variability among cytokine expression and secretion when compared with cell line groups [46], as seen here.

With regards to the effect of MTX on cytokine modulation, there have been conflicting reports. Early reports by Loetscher et al. claimed that MTX is ineffective at mediating IL-8 production in RA [47], while Kraan et al. reported decreased IL-8 in synovial fluid after MTX treatment [48]. Similarly, Nishina et al. [49] recently reported MTX effectively reduced IL-6 plasma levels in RA patients, while Inoue et al. [50] claimed that MTX did not have an inhibitory effect on IL-6 production by RA synovial cells. Previous studies conducted by our group found MTX delivery alone to be inconsistent, providing further evidence that the therapeutic effects of MTX may not be manifested in changes in the cytokine milieu [25].

The cytokine results portrayed in Figs. 2, 3, 4 and 5 in response to MTX alone are reflective of the variable response observed among RA patients. In addition to unpredictable efficacy, MTX is associated with a number of severe, dose-dependent side effects, limiting the tolerable dosage level. Several reports advocate for combination therapy of DMARDs, particularly MTX, with biologic therapies. For example, a report by Goekoop-Ruiterman et al. [51] claimed increased clinical improvement in early stages of diseases progression with combination therapies. Likewise, claims of increased efficacy of low-dose MTX combined with alternative therapies, such as phosphodiesterase type 3 inhibitor cilostazol, have also been reported [52]. Indeed, primary RASF cells showed a significant response in IL-6 levels to treatment with NP-ODN-MTX and NP-ODN, but not NP-MTX or MTX alone. The primary cell model also resulted in a significant reduction of IL-8 in response to NP-ODN, NP-MTX, NP-ODN-MTX, and MTX in comparison with an untreated control; however, only NP-ODN-MTX resulted in a significant decrease in comparison with just ODN delivery alone. These results suggest that decoy ODN has the ability to act alone, as well as to enhance efficacy of DMARD MTX when delivered in combination. NP provide a delivery vehicle to safely enhance cellular uptake of ODN, as well as encapsulate and carry MTX to the required site of action.

## Conclusion

In this study, we obtained results furthering our claim that NP can be used to effectively deliver nucleic acid-based drugs. Furthermore, we showed the combination of NF-κB decoy ODN and DMARD MTX resulted in reduction in inflammatory cytokines in both cell line and primary RASF models of RA in more instances than treatment with either therapy individually. To our knowledge, this is the first report investigating combination therapy of MTX with a decoy ODN. While NP have been used to administer both MTX and ODN separately in previous studies, this is the first time we have attempted to combine these two therapies and report successful modulation of inflammatory proteins in RA in vitro models [24, 25]. Incorporating in vivo testing is necessary to determine both safety and efficacy of PSA–TMC loaded with ODN and MTX, but this preliminary in vitro investigation provides strong evidence to support future studies.

## Abbreviations

ANOVA: Analysis of variance
CF: Cystic fibrosis
DMARD: Disease-modifying anti-rheumatic drug
DMEM: Dulbecco’s modified Eagle’s medium
ELISA: Enzyme-linked immunosorbent assay
EPR: Enhanced permeation and retention
FBS: Fetal bovine serum
HFLS: Human fibroblast-like synoviocytes
HPLC: High-performance liquid chromatography
IκB: Inhibitor of NF-κB
IL: Interleukin
IRB: Institutional Review Board
MTX: Methotrexate
NF-κB: Nuclear factor kappa-light chain enhancer of activated B cells
NP: PSA–TMC nanoparticles
ODN: Oligonucleotide
PBS: Phosphate-buffered saline
PEG: Polyethylene glycol
PEI: Poly(ethyleneimine)
PSA: Polysialic acid
RA: Rheumatoid arthritis
RES: Reticuloendothelial system
SCO: Scrambled oligonucleotide
SD: Standard deviation
TMC: *N*-trimethyl chitosan
TNFα: Tumor necrosis factor alpha
TPP: sodium tripolyphosphate
VEGF: vascular endothelial growth factor
